# The Hospital World

**Published:** 1911-02-18

**Authors:** 


### The Hospital World.

### EARL CAWDOR AND THE LONDON HOMŒOPATHIC HOSPITAL.

The hand of death has borne heavily upon the London Homœopathic Hospital during the last few months. It was only a short time since the hospital had to mourn the loss of two of its Vice-Presidents, viz. the Earl of Egmont and Lord Calthorpe, followed later by the loss by death of its Senior Consulting Physician, Dr. Dyce Brown. Within the next few days Mr. Joseph Henry Houldsworth, who was for many years an earnest and devoted supporter of the hospital, died. The hospital has now to report with profound sorrow the loss of the honoured and revered Treasurer, the Earl Cawdor. Lord Cawdor has been identified with the hospital as Teasurer since 1894, and during those years there has been no one better known in connection with the institution or more representative of it than his lordship. For more than seventeen years he zealously filled the post of Treasurer, and unceasingly continued to watch over the hospital's interest and its progress.

By his death the hospital and Homœopathy have sustained a loss which can hardly at present be fully estimated or realised.

Where such long continued services have been rendered it is superfluous to enter into further details, but two or three acts of immense importance to this hospital call for special remembrance at such a time as this.

In 1897, when Viscount Emlyn, he took the chair at a banquet at the Hôtel Cecil and raised a sum of £7,156 to complete the building fund of the present hospital building. In 1903 Lord Cawdor again presided at a dinner and raised £5,731 to replace the debt due to Capital Funds. Again in 1907 his lordship presided at the Hôtel Ritz and raised a Dinner Fund of £11,245 towards the New Wing Extension Fund; and in 1909 he had only just completed his appeal and raised £13,670 to complete the New Wing Extension Fund and build half the home for the nurses.

It is unnecessary to enlarge upon the important nature of these services, but during the long course of Lord Cawdor's connection with the hospital his advice and guidance of its affairs have been accompanied with advantages the value of which can hardly be over-estimated. The new Sir Henry Tyler Wing of the hospital was contemplated by him with much satisfaction, and while the Board must regret that its Treasurer has not been spared to witness the completion of the new wing in which he took so warm an interest, it must yet be most thankful to remember that it had his assistance and presence on the occasion of the laying of the corner stone by Sir George Truscott, Bart., on June 30, 1909, and most gratified to feel that by the completion of his Appeal Fund to complete the payment of the New Wing he may thus be said to have brought to its consummation the past successful career of the hospital and inaugurated that future for it which bears every promise of even larger public utility and prosperity.

Lamenting the loss of so loyal, so enthusiastic and so wise a Treasurer, the Board cannot but feel thankful that the hospital has been granted Lord Cawdor's powerful aid for so long a period. Members of the Board wish to record their sorrow at the death of their honoured and beloved Treasurer, and beg to offer to the Countess Cawdor and to the members of his family their heartfelt sympathy and condolence.

Frederick Archibald Vaughan Campbell, Earl Cawdor and Viscount Emlyn in the Peerage of the United Kingdom, and Baron Cawdor in that of Great Britain, was born on February 13, 1847. He died peacefully in his sleep on Wednesday, February 8 last, after an illness of some weeks' duration. It is unnecessary here to detail his public career, to which so much space has been devoted by the Press during the past week. His association with hospitals and charities may, however, be referred to very briefly. In addition to his very able work as Treasurer of the London Homœopathic Hospital, he was president of the Carmarthen County Infirmary and interested in the hospitals at Pembroke and Nairn, where his country seats were situate. He was Lord Lieutenant of Pembrokeshire from 1896, a Justice of the Peace for the counties of Carnarvon and Pembroke, Deputy-Lieutenant of the counties of Nairn and Inverness, President of the Pembroke Territorial Force Association from 1908, head of the Carnarvon Artillery Militia for ten years, an Ecclesiastical Commissioner since 1880, and an Honorary Commissioner in Lunacy from 1886 until 1893.

---

Mr. Holford Harrison, the re-elected treasurer of the Liverpool Royal Infirmary, received a special compliment from the Lord Mayor at the recent annual meeting on the skill he showed in the difficulties which beset his post.

The annual report of the Plymouth Royal Eye Infirmary contains an interesting reference to the late secretary, Mr. Edmund Pridham, a very well known figure to the hospital's friends. Indeed, Mr. Pridham's connection with the Royal Eye Infirmary had lasted for fifteen years, and during six years of that time he had been honorary secretary, maintaining a constant enthusiasm for his work. When he retired from this post it was only to take up the work of a committeeman, and thus for nine years he was content to do the arduous routine work that properly belongs to this office. Within a short time of his death Mr. Pridham's successor in the honorary secretaryship, Mr. Reginald Woollcombe, sent in his resignation, and thus the institution last year suffered a very serious loss in the personnel of its staff. Mr. Woollcombe retires, with an illuminated address, on to the committee, and the new secretary, Mr. Ernest Howard, has just performed one of his first formal duties, the presentation last week of the hospital's annual report. Mr. Edmund Pridham's death removes, by the way, one of the three oldest supporters of the institution, whose memories go back to the early days when the struggle for existence was a difficult problem for the hospital.

### Elections and Appointments.

Sir Charles G. Boxall, K.C.B., Mr. George Beal, Mr. T. Billing, Colonel Cavenagh, Mr. J. W. Penfold, Mr. F. R. Richardson, Mr. H. H. E. Scatliff, Mr. Austin Smith, the Rev. A. D. Spong, Mr. Wilson Stuckey, and Mr. H. Montague Williams have been re-elected as the committee of management of the Brighton, Hove, and Sussex Throat and Ear Hospital.

The Mayor of Brighton, the Vicar of Brighton, Alderman Henry Abbey, J.P., Mr. S. Copestake, Mr. C. Challis, the Rev. J. B. Figgis, Mr. William Foster, Mr. Walter Harrison, Mr. J. W. Howlett, J.P., the Mayor of Hove, the Vicar of Hove, the Chevalier Kuhe, Mr. G. W. E. Loder, J.P., Mr. T. J. Usher Robins, Colonel G. T. Shaft, and Mr. D. James have been elected Governors of the Brighton, Hove, and Sussex Ear and Throat Hospital.

